# Can you know before you go? Information about disability accommodations on US hospital websites

**DOI:** 10.1002/jhm.13477

**Published:** 2024-08-07

**Authors:** Allison Kannam, Carol Haywood, Megan A. Morris, Lynn Huang, Tracey Singer, Gurasees Bajaj, Aijalon Muhammad, Tara Lagu

**Affiliations:** ^1^ Northwestern University Feinberg School of Medicine Chicago Illinois USA; ^2^ Department of Medical Social Sciences Northwestern University Feinberg School of Medicine Chicago Illinois USA; ^3^ Division of General Internal Medicine, Department of Medicine University of Colorado Anschutz Medical Campus Aurora Colorado USA; ^4^ Center for Health Services and Outcomes Research, Institute of Public Health and Medicine Northwestern University Feinberg School of Medicine Chicago Illinois USA; ^5^ Spelman College Atlanta Georgia USA; ^6^ Division of Hospital Medicine, Department of Medicine Northwestern University Feinberg School of Medicine Chicago Illinois USA

## Abstract

**Background:**

People with disability (PWD) face challenges accessing healthcare. Websites are a public‐facing resource that can help PWD determine if a hospital can accommodate their needs, yet few studies have described whether hospital websites contain adequate accommodation information.

**Objective:**

To characterize the extent to which information about disability accommodations is available on US hospital websites.

**Methods:**

We manually reviewed hospital websites using a structured extraction form. We used the Centers for Medicare and Medicaid Services' Hospital General Information Data set to identify a stratified random sample of 600 nonspecialty hospitals in the United States. We excluded hospitals that shared a website with a previously reviewed hospital for a final sample of 445. We recorded (1) content about specific disability accommodations (in 11 predetermined categories); (2) descriptions of hospital policy mentioning disability; and (3) the point of contact to obtain more information about accommodations.

**Results:**

About two‐thirds (65.6%) of sampled hospitals were acute care hospitals (vs. 34.4% critical access); 53.5% had 26–299 beds. Overall, 73.7% websites had information about accommodations; of these, 36.3% had information solely within hospital policies. Of the 47.0% websites with accommodation information beyond hospital policies, the mean number of accommodations listed (excluding policy statements) was 2.37 (of 11 possible). Hospitals with 300+ beds had higher odds of listing any nonpolicy accommodations than those with 1–26 beds (odds ratio = 2.768, *p* = .02). Less than half (40.5%) hospitals listed a contact person.

**Conclusions:**

Information about disability accommodations is sparse on hospital websites. Comprehensive and actionable communication about accommodations is needed to better protect PWD's rights to accessible healthcare.

## Visual Abstract

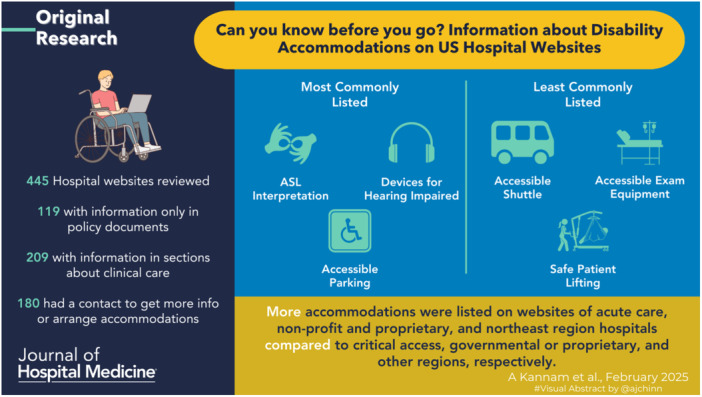

## INTRODUCTION

Approximately 27% of US adults have some type of disability.^1^ The Americans with Disabilities Act of 1990^2^ (ADA) and Section 504 of the Rehabilitation Act of 1973^3^ require healthcare organizations to provide people with disability (PWD) full and equal access to healthcare, which includes ensuring effective communication and making reasonable modifications to accommodate PWD. Reasonable accommodations can include height‐adjustable tables for patients with mobility disabilities, hand‐held amplifiers for patients with hearing disabilities, large print materials for patients with vision disabilities, or communication boards for patients with speech disabilities. Despite mandates, accommodations are often inconsistently available, resulting in physical^4^, ^5^, ^6^, ^7^ and communication^8^, ^9^, ^10^, ^11^ barriers to care.^12^, ^13^

Websites are a public‐facing resource that can provide patients with vital information about accommodations prior to presenting for care. Websites can provide information about hospital or physician quality,^14^, ^15^ the risks and benefits of procedures,^16^, ^17^ and more to allow potential patients to make informed decisions about their care.

With the exception of one study focused on language services in hospitals,^18^ there is little research focused on how often hospitals provide information to prospective patients about what accommodations are available or how to request them. Existing work in this area have described the accessibility level of the websites themselves^19^, ^20^ but has not documented the content of websites regarding accommodations available at the clinical sites they represent. We aimed to characterize the extent to which information about disability accommodation is available on US hospital websites.

## METHODS

### Website sampling

**Figure 1:**
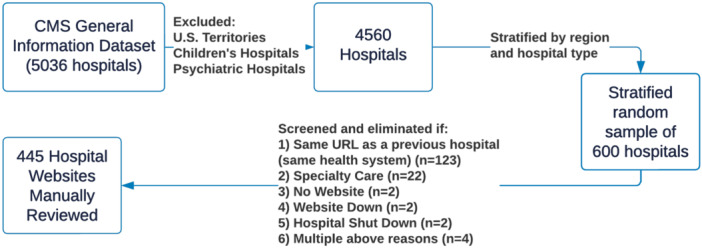
Sampling and screening process to determine hospitals for review. CMS, Centers for Medicare and Medicaid Services.

We identified a random sample of hospitals included in the Centers for Medicare and Medicaid Services' (CMS) Hospital General Information data set, which includes all hospitals registered with Medicare, in June 2022. We first excluded hospitals outside the 50 US states and DC, as well as psychiatric hospitals and children's hospitals (we aimed to exclude specialty care hospitals, and the data set only identifies these two types of specialty care hospitals). Next, we stratified the remaining 4560 hospitals by geographic region (Midwest; Northeast; South; West) and hospital ownership (governmental or tribal; nonprofit; proprietary; or physician‐owned). We identified the proportion of hospitals in the data set within each category (e.g., the proportion of nonprofit Midwestern hospitals). Then, we identified a random sample of 600 hospitals (13.2%) that was proportionally representative of hospitals in the overall data set (Figure 1).

### Creation of study materials

The research team, including experts in disability and health services research, created an extraction form and associated data collection protocol a priori. The first author reviewed websites from 20 random hospitals from the CMS Hospital General Information data set, adding measures to the protocol as needed. Next, two researchers reviewed the same 10 websites using the draft protocol, refined the definitions of each measure, and updated the protocol. After reviewing additional websites and comparing their entries line‐by‐line to resolve discrepancies, the researchers determined adequate consensus had been reached, and the protocol was finalized (Appendix A). Two additional coders were trained by reviewing the protocol; they each independently reviewed eight websites to compare with the first author and clarify any points of confusion until the protocol was consistently applied.

The extraction form included the following domains: (1) the presence of content about the hospitals' accommodations for patients with mobility, hearing, vision, and speech disabilities (e.g., American Sign Language (ASL) interpreters, communication aids, accessible exam room equipment); (2) the presence of hospital policy statements (Nondiscrimination Statements, Patient Rights Statements, Website Accessibility Statements, and Effective Communication Policies; hereafter “Policy Statements”) containing information about accommodations; and (3) the point of contact for patients to obtain more information about accommodations (Appendix A). Presence of content about accommodations and policy statements were recorded as binary variables (yes = 1, no = 0). To focus on well‐defined, commonly requested accommodations, we included accommodations that improve accessibility for patients with mobility, hearing, vision, and speech disabilities. We extracted the number of staffed beds per hospital from the American Hospital Association's Data Hub (ahadata.com). In addition to geographic location and hospital ownership (used for stratified sampling), we extracted hospital type from the CMS Hospital General Information data set (critical access vs. acute care). A Critical Access Hospital is a designation assigned by CMS with criteria including rural location and location more than 35 miles from the nearest hospital. These hospitals typically have 25 or fewer inpatient beds (with exceptions).

### Data collection

Four trained coders reviewed hospital websites independently between July 2022 and May 2023. Coders searched the hospital's name on Google to identify the website URL and used the physical address listed in the data set to confirm it was the correct site. Coders then navigated the websites organically. That is, they did not follow a standardized process of clicking through the pages of the website to look for information on the extraction form, but rather navigated it as a patient might, clicking tabs that appeared relevant to the information on the form. Next, they searched the following words in the website's search bar to confirm relevant information was not missed: disability, accessibility, accommodation, impairment. If there were areas of uncertainty in the review process, coders left comments throughout the form for resolution by the first author, and in some cases, consensus among the author team.

We wanted this work to reflect information that is shared in patient‐centered areas of the website and intended for patients to gather information and make decisions, so we limited our analysis of Nondiscrimination Statements, Patient Rights Statements, and Website Accessibility Statements to the contact information included (most of the policy documents were very similar across websites, appeared to be legally required, and did not seem to have a primary purpose of communicating information to patients). For example, accommodations such as ASL interpreter services were only counted if they appeared on the website in a location other than the aforementioned policy statements. We did not include the contents of policy statements because they would overinflate certain variables due to the inclusion of ASL interpretation and low‐vision accommodation notices as examples in standard Nondiscrimination Statement language. We only recorded policy statements as present if they explicitly mentioned disability accommodations and only counted contact information if it was intended for patients to get more information about accommodations or request them. Contact information intended to file complaints or grievances was excluded; such contact information was most common in Nondiscrimination Statements.

### Analysis

We used descriptive statistics to summarize hospital characteristics and prevalence of accommodations. To define “Accommodation Information” for analysis, we recorded the presence of policy statements including accommodation information (Appendix A, Extraction Form Column: AM‐AP) as well as information about specific accommodations described outside policy statements in any of the categories of the extraction form (Appendix A, Extraction Form Columns: V‐Z and AA‐AF). Next, we calculated a cumulative sum of all accommodation information (in and outside of policy statements) which we defined as the “Number of Total Accommodations.” We then further examined how many sites included “Nonpolicy Accommodation Information,” that is, accommodation information provided outside policy statements (Appendix A, Extraction Form Columns: V‐Z and AA‐AF) which we summed into “Number of Nonpolicy Accommodations.” To clarify, if a website had accommodation information in a Nondiscrimination Statement and a Patient Rights Statement and also had patient‐facing pages about accessible parking and ASL interpretation, the number of total accommodations would be 4, and the number of nonpolicy accommodations would be 2.

After excluding websites with no accommodation information, we calculated the mean number of total accommodations and nonpolicy accommodations. We then calculated the mean number of total accommodations including sites without accommodations and used the nonparametric Kruskal–Wallis test to compare characteristics across groups. We used *χ* ^2^ tests to examine associations between the presence of contact information and hospital characteristics. We used a logistic regression to examine predictors of having 1+ nonpolicy accommodations, adjusted for the following hospital characteristics: hospital type, hospital ownership, geographic region, and number of hospital beds (1–25; 26–299; 300+).

We used qualitative content analysis^21^ to code data about hospital contacts (see Appendix B for the codebook). After coding was completed, we created variables to differentiate hospital contacts coded as “Disability‐Related” with those that were “Nondisability Related” (e.g., departments handling legal issues) and with those that did not list a contact person; the mean number of total accommodations was compared across these categories using the nonparametric Kruskal–Wallis test. All statistical analyses were performed using SAS V 9.4 (SAS Inc.).

## RESULTS

After screening the 600 sampled hospital websites and eliminating websites with the same URL as a previously reviewed website (e.g., same health system), additional specialty care hospitals not removed at the sampling stage (e.g., orthopedic hospitals, surgical hospitals), hospitals that no longer exist, and websites that were down or did not exist, we reviewed 445 hospital websites (74.2%, Figure 1). Most hospitals were acute care (65.6%) and 34.4% were critical access. Most were nonprofit hospitals (56.4%), followed by governmental or tribal (28.5%) and proprietary or physician‐owned hospitals (15.1%). Hospitals were spread across geographic regions (40.0% South, 28.8% Midwest, 20.2% West, and 11.5% Northeast) and varied in size (29.2% had 1–25 beds, 53.5% had 26–299 beds, and 17.3% had 300+ beds).

Overall, 73.7% (*n* = 328) of websites had any accommodation information. Of these websites, 36.3% (*n* = 119) had information exclusively in policy statements, and 63.7% (*n* = 209) included accommodation information in areas of the website that intended to convey information about clinical care to patients, which was less than half of the total number of websites reviewed (47.0%). The accommodations most commonly specified outside of policy statements were ASL Interpreter Services, Assistive Devices for Hearing Impairment, and Accessible Parking. The least commonly listed accommodations were Accessible Shuttles, Accessible Exam Room Equipment, and Safe Patient Lifting. Nondiscrimination Statements with accommodation information were available about half of the time (53.0%, *n* = 236) (Table 1).

**Table 1:** Prevalence of information about various accommodation types on hospital websites.

Content type	Hospitals (*n*, %) (*N* = 445)
Presence of policy statements with accommodation information	
Nondiscrimination Statement	236 (53.0)
Patient Rights Statement	140 (31.5)
Website Accessibility Statement	101 (22.7)
Effective Communication Policy	9 (2.0)
Prescence of accommodation information listed outside policy statements	
ASL Interpreter Services	179 (40.2)
Assistive Devices for Hearing Impairment	94 (21.1)
Accessible Parking^a^	56 (12.6)
Low Vision Accommodations	50 (11.2)
Patient Escort Services	27 (6.1)
Accessible Entrances^a^	22 (4.9)
Communication Aids for Speech Impairment	19 (4.3)
Accessible Restrooms^a^	18 (4.0)
Accessible Shuttles^a^	16 (3.6)
Accessible Exam Room Equipment^a^	9 (2.0)
Safe Patient Lifting^a^	6 (1.4)

Abbreviation: ASL, American Sign Language.

a Categories relevant to patients with mobility disabilities.

Less than half (40.5%, *n* = 180) of the websites (*N* = 445) had contact information listed for patients to get more information about accessibility or to arrange accommodations. Of the proportion that listed such contact information, the title or role of the contact person varied greatly (Table 2). Larger hospitals were more likely to list contact information: contact information was included for 24.4% of hospitals with 1–25 beds; 42.0% of hospitals with 26–299 beds; and 62.3% of hospitals with 300+ beds (*p* < .0001). Additionally, more acute care hospitals (*N* = 292) listed contact information (49.7%, *n* = 145) than critical access hospitals (22.9%, *N* = 153, *n* = 35, *p* < .0001).

**Table 2:** Title categories of hospital contact persons.

Title category	Hospitals, *n* (%) (*N* = 180)
Administrator or provider	22 (12.2)
Civil Rights or DEI	15 (8.3)
Compliance, risk, or other legal	30 (16.7)
Disability‐related	30 (16.7)
Language‐specific	24 (13.3)
Patient‐focused	24 (13.3)
Unspecified	35 (19.4)

Abbreviation: DEI, diversity, equity, and inclusion.

Of websites listing any accommodations, the mean number of total accommodations was 2.99, and the mean number of nonpolicy accommodations was 2.37 (out of 11 possible). Table 3 presents the mean number of total accommodations, both inclusive and exclusive of zero, as well as the mean number of nonpolicy accommodations, listed on websites by hospital characteristic and contact information type. Of note, acute care hospitals and hospitals with a disability‐related contact person had significantly more nonpolicy accommodations listed on average; critical access hospitals, governmental or tribal hospitals, and hospitals without a contact person listed significantly fewer (*p* < .0001; *p* < .0001; *p* < .0001, respectively). In addition, adjusting for other hospital characteristics, hospitals with 300+ beds had higher odds of listing any nonpolicy accommodations than those with 1–26 beds (odds ratio [OR] = 2.768, *p* = .02); Northeastern hospitals had higher odds of doing so than Western hospitals (OR = 3.238, *p* = .006). Conversely, critical access hospitals had decreased odds of listing any accommodations than acute care hospitals (OR = 0.476, *p* = .02), and proprietary or physician‐owned hospitals had decreased odds of doing so than nonprofit hospitals (OR = 0.483, *p* = .02) (Appendix C).

**Table 3:** Mean number of accommodations by hospital characteristic.

Hospital characteristic	Mean number of total accommodations listed (*N* = 445)	Mean number of total accommodations among hospitals with any (nonzero) (*N* = 328)	Mean number of nonpolicy accommodations (*N* = 445)
Region	(*p* = .0035)	(*p* = .2384)	(*p* < .0001)
South	2.02	2.72	0.83
Midwest	2.27	3.16	1.23
West	1.92	2.98	1.01
Northeast	3.18	3.44	1.98
Hospital type	(*p* < .0001)	(*p* < .0001)	(*p* < .0001)
Acute care	2.72	3.2	1.39
Critical access	1.21	2.34	0.59
Hospital ownership	(*p* < .0001)	(*p* = .0012)	(*p* < .0001)
Nonprofit	2.54	3.16	1.4
Governmental or tribal	1.37	2.35	0.7
Proprietary or physician‐owned	2.55	3.22	0.82
Role of hospital contact person	(*p* < .0001)	(*p* = .003)	(*p* < .0001)
Disability‐related contact person	4.07	4.07	1.9
Nondisability‐related contact person	3.23	3.26	1.65
No contact person listed	1.42	2.52	0.72

## DISCUSSION

Websites are a key entry point for PWD to access information about available accommodations and request accommodations. Unfortunately, we found that accommodation information is sparse and incomplete, which aligns with the barriers^4^, ^5^, ^6^, ^7^, ^8^, ^9^, ^10^, ^11^ PWD experience in obtaining disability accommodations federally mandated by the ADA and Section 504 of the Rehabilitation Act.^2^, ^3^ A quarter of hospitals in our sample had no information about accommodations and 60% of hospitals failed to provide contact information that would allow patients to call the hospital with questions or requests. Websites that included accommodation information generally provided few details.

Less than half of sampled hospitals included accommodation information in areas of their websites intended to convey information to patients about clinical care; a quarter only mentioned accommodations in policies such as Nondiscrimination Statements or Website Accessibility Statements written to satisfy legal and regulatory requirements and lacking patient‐friendly language. These statements are required by Section 1557 of the Patient Protection and Affordable Care Act^22^ and Section 508 of the Rehabilitation Act,^23^ respectively. Only including accommodation information in the fine print of a legal notice suggests that hospitals are meeting the legal requirements but are perhaps missing the spirit of the legislation.

To further contextualize our results, we examined how often a patient with a specific disability type would be able to determine available accommodations from websites. For example, there are six mobility‐related accommodations relevant to a patient using a wheelchair (footnote “a” in Table 1), but no hospital websites mentioned all six accommodations, and only 14 hospitals listed ≥3 total accommodations. Accommodations for hearing impairment, including ASL interpreters and assistive devices, were the most frequently listed accommodations on websites, but were only available on 40.2% and 21.1% websites, respectively. Notably, ASL interpretation was often presented alongside other language interpretation services, which have additional legal protections and may be more widespread on hospital websites.^18^ Overall, these patterns are unsurprising, given that the ADA is primarily enforced reactively following lawsuits after accommodations are denied to PWD, and cases of discrimination in healthcare are primarily related to effective communication for patients with hearing disability.^24^ Of note, in May 2024, a final rule was issued to update the requirements of Section 504 of the Rehabilitation Act. For example, recipients of federal funding must have available at least one accessible examination table and weight scale.^25^ Such updates may provide additional urgency for systems to proactively secure necessary accommodations for patients and communicate their availability.

Larger hospitals had greater odds of listing accommodations than smaller hospitals and were more likely to list contact information. This finding aligns with a similar study on information about language services on hospital websites, which found larger bed‐size, higher revenue, and more admissions to be associated with providing language services information.^18^ Critical access hospitals had fewer accommodations listed on average and were less likely to list contact information. This is particularly notable because limitations in healthcare access in rural areas^26^ may make prior knowledge of accommodations even more crucial in these settings.

The type of contact information shared on hospital websites, if present, varied greatly. Many hospitals' contact persons had titles such as “Risk Coordinator,” which may indicate that the position is focused on avoiding lawsuits rather than championing equitable patient care. Of note, the Affordable Care Act requires a “Compliance Coordinator” within covered entities with greater than 15 employees, which may drive some hospitals' decision to maintain a position with this title.^22^ Given the increasing body of research around many physicians' unwelcoming attitudes around treating PWD,^27^, ^28^ however, patients may be understandably dissuaded from contacting the “Risk Coordinator,” because of fear of being labeled as a troublesome patient.

In a small proportion of the hospitals, the contact person appropriately had a disability‐focused professional title such as “ADA Coordinator” (a position required by Title II of the ADA for public entities with greater than 50 employees).^2^ These hospitals also had a significantly higher mean number of accommodations listed on the website than hospitals with nondisability‐related contact persons or no listed contact person. Although a designated staff person dedicated to ensuring accessibility for PWD may lead to better communication about accommodations, hiring an ADA coordinator may not be sufficient to ensure that accommodations are provided; previous research suggests that these roles are often ill‐defined and there is a very wide range across hospitals of what the role actually encompasses.^29^ Additionally, qualitative research involving disability coordinators suggests that even when a dedicated staff person exists, operationalizing the delivery of accommodations requires executive leadership buy‐in and support.^30^

Notably, because of a lack of standards for hospital website content regarding accommodations and because website content may not reflect the actual care being delivered, our study cannot be used to definitively determine the accessibility level of the hospitals reviewed. Unfortunately, patients may see an accommodation listed on the website and find when they arrive at the hospital that it does not exist, is of inadequate quality, is unfamiliar to staff, or is difficult to procure. Hospitals in our study may also have accommodations that are not listed on their website, especially more basic accommodations such as accessible parking, accessible entrances, and accessible restrooms. Therefore, this work reflects the extent to which hospitals use their websites to convey a culture of acceptance, welcoming, and customer service to a historically marginalized group who are often discriminated against in healthcare settings. Based on our results, there appears to be significant room for growth.

Our findings have important implications. Patients may have limited choice when seeking hospital care based on where they live, where their outpatient doctors are affiliated, or what services a given hospital provides. As such, providing adequate information about accommodations may not influence *if* a patient presents for care as much as how adequately their needs are met *when* they present for care. Therefore, the onus of responsibility must be on hospitals to ensure patients receive equitable care. In addition, there are opportunities for future research focused on how PWD interact with hospital websites before seeking care and their preferences for content. This would aid in the development of best practices for communicating accommodation information on websites and may assist hospital staff with doing so more effectively.

Our study also has several limitations. Our extraction form focused on four main types of disabilities—mobility, hearing, vision, and speech—and was not designed to reflect accommodation information on other disability types including mental, cognitive, or intellectual disabilities. Additionally, despite every effort to systematize our methodology, reviewing websites is an inherently subjective process. Given wide diversity of needs and preferences among PWD, our review may over‐ or undercharacterize what a PWD would draw from a website if reviewing for their own benefit. Moreover, our reviews reflect a point‐in‐time snapshot of a website and websites can change over time.

### CONCLUSIONS

Improved communication is needed regarding disability accommodations in the hospital setting. Too many hospitals do not list accommodation information on their websites or bury it within legally mandated policy statements. The burden of accessible care cannot fall solely on PWD; hospital leaders must remain committed to serving the largest minority group in the country. Listing basic information about disability accommodations on their websites is a low‐cost, straightforward place to start.

## CONFLICT OF INTEREST STATEMENT

The authors declare no conflict of interest.

## APPENDIX A WEBSITE REVIEW PROTOCOL

**Instructions**

Once a hospital has been chosen, we will use the name and address to locate the hospital's website through Google. We will first scroll through the website to see if there are intuitive places where accessibility and accommodation information might be—a “patient information” or “patient services” tab, for example. After reviewing on our own, we will use the website search bar on the top right (if one exists) to search the following four terms: disability, accessibility, accommodation, and impairment. Note that this is within the website, not Google. This may bring up additional web pages we missed with pertinent information.

Global Notes:

- We are not interested in information about accommodations for new or existing employees and hiring—we are specifically focused on patients/family members/visitors of a hospital.
- For Nondiscrimination Statements, Patient Rights Statements, and Website Accessibility Statements, we will record whether or not they exist and whether or not they contain information about accommodations, but WILL NOT extract actual information about accommodations to be recorded elsewhere in the form.
- However, we will always record contact information specific to accommodations/accessibility, regardless of where on the website it is posted (i.e., we will extract contact information from policy statements).
- Highlight a cell yellow if you were unsure how to record the data. This will flag it for future discussion and decision.

**Data gathered on extraction form**

**Table:**

Extraction form column	Variable name	Format for documenting data	Inclusions, exclusions, or additional instructions (if applicable)
A–E, G–K, M	Facility ID, facility name, address, city, state, zip code, county name, phone number, hospital type, hospital ownership, emergency services	N/A	Note: These fields were already present from the CMS General Information Data set.
F	Region	N/A	Recoded by research team from “State” and used for stratified sampling Midwest = IA, IL, IN, KS, MI, MN, MO, ND, NE, OH, SD, WI Northeast = CT, MA, ME, NH, NJ, NY, PA, RI, VT South = AL, AR, DC, DE, FL, GA, KY, LA, MD, MS, NC, OK, SC, TN, TX, VA, WV West = AK, AZ, CA, CO, HI, ID, MT, NM, NV, OR, UT, WA, WY
L	Hospital ownership collapsed	N/A	Recoded by research team from “Hospital Ownership” and used for stratified sampling Government + Tribal + Department of Defense → Governmental or Tribal Proprietary + Physician → Proprietary or physician‐owned Voluntary nonprofit → Nonprofit
N	Date of website review	MM/DD/YY	
O	Reviewer initials	First Initial_Last Initial	
P	Excluded from review	1 = Yes 0 = No	
Q	Reason for exclusion	Write‐in text	Options include: Duplicate, specialty care (e.g., orthopedic, surgical, heart hospital) hospital shut down, website down, no website. Duplicate URLs are highlighted by conditional formatting in the Excel sheet.
R	Actual hospital name	Write‐in text	Only write if different than what is listed in Column B, otherwise leave blank.
S	URL	Write‐in text	The URL should be to the homepage of the hospital or health system that it is a part of. If your search brings you to the “Location and Directions” page for a given hospital, go to the health system homepage to begin your search—this will ensure that future hospitals in this health system are flagged as duplicates. In a small number of select hospitals determined on a case‐by‐case bases, the hospital and health system homepage may be different.
T	Number of staffed beds	Number	Look up on ahadata.com
U	Does hospital or health system have outpatient specialty care	1 = Yes 0 = No	Two or more specialties is required to select “Yes.” For our purposes, outpatient specialty care includes services such as cardiology, pulmonology, neurology, GI, and so on, and excludes ER, primary care, lab services, rehab therapy or hospice, imaging, and so on.
V	Accessible parking	1 = Yes (website has mention of this) 0 = No	
W	Accessible shuttles	1 = Yes 0 = No	
X	Accessible entrances	1 = Yes 0 = No	
Y	Accessible restrooms	1 = Yes 0 = No	
Z	Patient escort services	1 = Yes 0 = No	
AA	Assistive devices for hearing impairment	1 = Yes 0 = No	Including but not limited to devices such as TDDs, Pocket Talkers, CART
AB	ASL interpreter services	1 = Yes 0 = No	
AC	Accommodations for accessing information with low vision	1 = Yes 0 = No	Including but not limited to accommodations such as large print, audio, and accessible electronic formats.
AD	Accessible exam room equipment	1 = Yes 0 = No	Including but not limited to accommodations such as adjustable table, weight scales for use with a wheelchair.
AE	Safe patient lifting	1 = Yes 0 = No	Including but not limited to lifts, staff trained in lifting.
AF	Communication aids for speech impairment	1 = Yes 0 = No	Including but not limited to communication aids such as writing materials, typewriters, computers, flashcards, alphabet boards, communication boards.
AG	Other accommodations	Write‐in text	
AH	Specific instructions to get accommodations	Copy‐and‐paste text from website	Copy and paste in the minimum amount of information needed to give context, can copy and paste from multiple places on website, blank if nothing. Examples include: call ahead at x number, speak to nurse, and so on.
AI	Contact information	1 = Yes 0 = No	This can be gathered from any part of the website (including nondiscrimination notices, patient rights, etc.) Includes: Contact information for questions about accommodations or to request accommodations. Excludes: contact information specifically intended for complaints or grievances.
AJ	Name and title of person or office to contact	Copy‐and‐paste text	
AK	Contact information	Copy‐and‐paste text	Phone number and/or email, include both if both are present, blank if nothing.
AL	Specific disability categories mentioned	Write‐in text	Website mentions that resources are available for a category (or multiple) of patients with disabilities, separate from and regardless of any specific accommodations mentioned on the website. For example, “We provide accommodations for people with mobility impairments.” Including but not limited to mobility, vision, hearing, speech, intellectual, cognitive, and mental.
AM	NDS with information about accommodations	1 = Yes 0 = No	Exclude if the NDS does not state anything about accommodations/accessibility, such as if it simply states that the hospital does not discriminate against PWD. Do not extract data from the NDS to include in Columns V through AG.
AN	Accommodation information in patient rights statement	1 = Yes 0 = No	Exclude if there is a Patient Rights Statement that does not state anything about accommodations or accessibility for PWD, or if it states a right to “good communication” but includes no specifics about accommodations. Do not extract data from the Patient Rights Statement to include in Columns V through AG.
AO	Language about effective communication Policy	1 = Yes 0 = No	Example Template: https://www.hhs.gov/civil-rights/for-providers/clearance-medicare-providers/auxiliary-aids-persons-disabilities/index.html
AP	Website accessibility statement or widget	1 = Yes 0 = No	Also count this as yes if the webpage has a “widget” that allows you to adjust accessibility settings.
	Notes	Write‐in	Usually can be blank, only to be used for any important contextual information about the process of reviewing not captured elsewhere. If anything highlighted yellow for cross‐checking, can add details there.

Abbreviations: ASL, American Sign Language; CART, communication access real time translation; ER, emergency room; GI, gastrointestinal; NDS, Nondiscrimination Statement; PWD, people with disability; TDD, telecommunication devices for the deaf.

## APPENDIX B CONTACT INFORMATION CODEBOOK

This codebook outlines the contact information titles included in each code.

**Administrator or Provider:** Admissions, Case Management, Scheduling, Nursing, House Supervisor, HR, Senior Vice President of Finance, Social Worker, Volunteer Services.

**Civil Rights and DEI:** Civil Rights Coordinator, Nondiscrimination Coordinator, Section 1157 Coordinator, Center for Equity of Care, Diversity and Inclusion Department.

**Compliance, Risk, or Other Legal:** Compliance Officer or Department, Compliance and Privacy Officer, Equity Compliance Coordinator, Ethics and Compliance Officer, Grievance Hotline/Officer, Legal Compliance Officer, Risk Officer or Coordinator, Appeals and Correspondence Department, Corporate Responsibility, HHS, Office of General Counsel, Quality Department.

**Disability‐Related:** 504 Coordinator, ADA Coordinator, Assistive Device Point Person, Commission for the Deaf and Hard of Hearing, Communication Access, Disability Resource Center, Disability Access Officer, Disability Program Manager, ASL, and Deaf Interpretation Services.

**Language‐Specific:** Interpreter Services, Language Access Coordinator, and Language Services.

**Patient‐Focused:** Chief Patient Experience Officer, Coordinator of Patient Resources, Patient Advocate, Patient and Family Experience, Patient Relations, and Patient Care Coordinator.

**Unspecified Title:** Name, phone, or email address with no title listed.

*Notes*:

For titles that spanned more than one code in which one was disability‐related (*n* = 17) (e.g., “Chief Compliance Officer ADA/504 and Section 1557 Coordinator,” “Risk Officer/ADA Coordinator”), the title was coded under “Disability‐Related.” Given the focus of this study, any mention of disability in the title was of interest. Titles (*n* = 3) such as “Compliance Officer and Civil Rights Coordinator” that spanned the “Compliance” and “Civil Rights” categories were coded as “Civil Rights,” as this designation is more specific than “Compliance.”

## APPENDIX C CHARACTERISTICS ASSOCIATED WITH 1+ NONPOLICY ACCOMMODATIONS ON HOSPITAL WEBSITE, LOGISTIC REGRESSION

Table C1.

**Table C1:** Odds of 1+ versus 0 nonpolicy accommodations by hospital characteristic.

Hospital characteristic	OR	95% CI	*p* Value
Hospital type			
Acute care	Reference		
Critical access	0.476	(0.25, 0.90)	.02
Hospital Ownership			
Nonprofit	Reference		
Proprietary or physician‐owned	0.483	(0.27, 0.88)	.02
Governmental or tribal	0.676	(0.41, 1.10)	.012
Number of hospital beds			
1–25	Reference		
26–299	1.515	(0.79, 2.91)	.21
300+	2.768	(1.21, 6.34)	.02
Region			
West	Reference		
South	0.753	(0.43, 1.31)	.31
Midwest	1.1	(0.61, 1.98)	.75
Northeast	3.238	(1.41, 7.46)	.006

Abbreviations: CI, confidence interval; OR, odds ratio.
